# Adverse childhood experiences and associations with health-harming behaviours in young adults: surveys in eight eastern European countries

**DOI:** 10.2471/BLT.13.129247

**Published:** 2014-06-19

**Authors:** Mark A Bellis, Karen Hughes, Nicola Leckenby, Lisa Jones, Adriana Baban, Margarita Kachaeva, Robertas Povilaitis, Iveta Pudule, Gentiana Qirjako, Betül Ulukol, Marija Raleva, Natasa Terzic

**Affiliations:** a Centre for Public Health, Liverpool John Moores University, 15–21 Webster Street, Liverpool L3 2ET, England.; b Department of Psychology, Babes-Bolyai University, Cluj-Napoca, Romania.; c Serbsky National Research Center for Social and Forensic Psychiatry, Moscow, Russian Federation.; d Department of Clinical and Organizational Psychology, Faculty of Philosophy, Vilnius University, Vilnius, Lithuania.; e Centre for Disease Prevention and Control, Riga, Latvia.; f Faculty of Public Health, University of Medicine, Tirana, Albania.; g Department of Social Paediatrics, Ankara University School of Medicine, Ankara, Turkey.; h University Clinic of Psychiatry, St Cyril and Methodius University, Skopje, The former Yugoslav Republic of Macedonia.; i Institute of Public Health, Podgorica, Montenegro.

## Abstract

**Objective:**

To evaluate the association between adverse childhood experiences – e.g. abuse, neglect, domestic violence and parental separation, substance use, mental illness or incarceration – and the health of young adults in eight eastern European countries.

**Methods:**

Between 2010 and 2013, adverse childhood experience surveys were undertaken in Albania, Latvia, Lithuania, Montenegro, Romania, the Russian Federation, The former Yugoslav Republic of Macedonia and Turkey. There were 10 696 respondents – 59.7% female – aged 18–25 years. Multivariate modelling was used to investigate the relationships between adverse childhood experiences and health-harming behaviours in early adulthood including substance use, physical inactivity and attempted suicide.

**Findings:**

Over half of the respondents reported at least one adverse childhood experience. Having one adverse childhood experience increased the probability of having other adverse childhood experiences. The number of adverse childhood experiences was positively correlated with subsequent reports of health-harming behaviours. Compared with those who reported no adverse experiences, respondents who reported at least four adverse childhood experiences were at significantly increased risk of many health-harming behaviours, with odds ratios varying from 1.68 (95% confidence interval, CI: 1.32–2.15) – for physical inactivity – to 48.53 (95% CI: 31.98–76.65) – for attempted suicide. Modelling indicated that prevention of adverse childhood experiences would substantially reduce the occurrence of many health-harming behaviours within the study population.

**Conclusion:**

Our results indicate that individuals who do not develop health-harming behaviours are more likely to have experienced safe, nurturing childhoods. Evidence-based programmes to improve parenting and support child development need large-scale deployment in eastern European.

## Résumé

**Objectif:**

Évaluer l'association entre des expériences négatives vécues pendant l'enfance (comme des abus, des négligences, des violences domestiques, la séparation des parents, l'usage de substances illicites, des maladies mentales ou l'incarcération) et la santé des jeunes adultes dans huit pays de l'Europe de l'Est.

**Méthodes:**

Entre 2010 et 2013, des enquêtes sur des expériences négatives vécues pendant l'enfance ont été menées en Albanie, en Lettonie, en Lituanie, au Monténégro, en Roumanie, en Fédération de Russie, dans l'ex-République yougoslave de Macédoine et en Turquie. On a compté 10 696 répondants, dont 59,7% de femmes, âgés de 18 à 25 ans. Une modélisation à variables multiples a été utilisée pour étudier les relations entre des expériences négatives vécues pendant l'enfance et les comportements mettant la santé en danger au début de l'âge adulte, notamment l'usage de substances illicites, l'inactivité physique et les tentatives de suicide.

**Résultats:**

Plus de la moitié des répondants ont déclaré au moins une expérience négative vécue pendant l'enfance. Le fait d'avoir vécu une expérience négative pendant l'enfance augmentait la probabilité de vivre d'autres expériences négatives pendant l'enfance. Le nombre d'expériences négatives vécues pendant l'enfance était positivement corrélé avec les signalements ultérieurs de comportements mettant la santé en danger. Par rapport aux personnes qui n'ont déclaré aucune expérience négative, les répondants qui ont déclaré au moins 4 expériences négatives vécues pendant l'enfance présentaient un risque significativement augmenté de nombreux comportements mettant leur santé en danger, avec des rapports de cotes variant de 1,68 (intervalle de confiance de 95%, IC: 1,32–2,15) pour l'inactivité physique, à 48,53 (IC de 95%: 31,98–76,65) pour les tentatives de suicide. La modélisation a indiqué que la prévention des expériences négatives vécues pendant l'enfance réduirait considérablement l'occurrence de nombreux comportements mettant la santé en danger au sein de la population étudiée.

**Conclusion:**

Nos résultats indiquent que les individus qui ne développent pas de comportements mettant leur santé en danger sont plus susceptibles d'avoir été protégés et éduqués pendant leur enfance. Les programmes fondés sur les faits visant à améliorer l'éducation des enfants et à soutenir leur développement nécessitent un déploiement à grande échelle en Europe de l'Est.

## Resumen

**Objetivo:**

Evaluar la relación entre las experiencias adversas en la infancia, por ejemplo, abusos, abandono, violencia doméstica y separación de los padres, consumo de sustancias, enfermedad mental o encarcelamiento, y la salud de los adultos jóvenes en ocho países orientales de Europa.

**Métodos:**

Entre 2010 y 2013 se llevaron a cabo encuestas sobre experiencias infantiles adversas en Albania, Letonia, Lituania, Montenegro, Rumania, la Federación de Rusia, la antigua República Yugoslava de Macedonia y Turquía. El número de encuestados fue de 10 696 - 59,7 % mujeres - de entre 18 y 25 años. Se empleó un modelo multivariado para examinar las relaciones entre las experiencias infantiles adversas y los comportamientos dañinos para la salud en las primeras etapas de la vida adulta, incluido el consumo de sustancias, la inactividad física y el intento de suicidio.

**Resultados:**

Más de la mitad de los encuestados informó de al menos una experiencia adversa en la infancia. Haber sufrido una experiencia adversa en la infancia aumentó la probabilidad de tener otras experiencias adversas en la infancia. El número de experiencias adversas en la infancia se correlacionó positivamente con los informes posteriores acerca de comportamientos dañinos para la salud. En comparación con aquellos que no comunicaron experiencias adversas, los encuestados que informaron de al menos cuatro experiencias adversas en la infancia presentaban un riesgo significativamente mayor de muchas conductas dañinas para la salud, con un cociente de posibilidades que variaba de 1,68 (intervalo de confianza del 95 %, IC: 1,32–2,15) para la inactividad física a 48,53 (IC del 95 %: 31,98–76,65) para el intento de suicidio. El modelado indicó que la prevención de experiencias adversas en la infancia podría reducir sustancialmente la incidencia de muchos de los comportamientos dañinos para la salud en la población de estudio.

**Conclusión:**

Nuestros resultados indican que los individuos que no desarrollan comportamientos dañinos para la salud tienen más probabilidades de haber tenido una infancia segura y educativa. Los programas basados en la evidencia para mejorar la crianza de los hijos y apoyar el desarrollo infantil necesitan un despliegue a gran escala en la región oriental de Europa.

## ملخص

**الغرض:**

تقييم الارتباط بين خبرات الطفولة السيئة – مثل سوء المعاملة أو الإهمال أو العنف المنزلي أو انفصال الأبوين أو تعاطي المخدرات أو الاعتلال النفسي أو الحبس – وصحة صغار البالغين في ثمانية بلدان أوروبية شرقية.

**الطريقة:**

أجريت بين عامي 2010 و2013 مسوح حول خبرات الطفولة السيئة في ألبانيا ولاتفيا وليتوانيا والجبل الأسود ورومانيا والاتحاد الروسي وجمهورية مقدونيا اليوغوسلافية السابقة وتركيا. وشارك في المسح 10696 شخصاً – 59.7 % منهم من الإناث – اللاتي تتراوح أعمارهن من 18 إلى 25 سنة. وتم استخدام النمذجة متعددة المتغيرات لتحري الارتباط بين خبرات الطفولة السيئة والسلوكيات الضارة بالصحة في البلوغ المبكر بما في ذلك تعاطي المخدرات والخمول البدني ومحاولة الانتحار.

**النتائج:**

أبلغ ما يزيد عن نصف المشاركين عن خبرة واحدة على الأقل من خبرات الطفولة السيئة. وازدادت مع التعرض لخبرة واحدة من خبرات الطفولة السيئة احتمالية التعرض لخبرات الطفولة السيئة الأخرى. وارتبط عدد خبرات الطفولة السيئة إيجابياً بالتقارير اللاحقة حول السلوكيات الضارة بالصحة. وتزايدت على نحو كبير خطورة اكتساب العديد من السلوكيات الضارة بالصحة لدى المشاركين الذين أبلغوا عن أربع خبرات على الأقل من خبرات الطفولة السيئة مقارنة بالذين لم يبلغوا عن خبرات سيئة، وتراوحت نسب الاحتمال من 1.68 (فاصل الثقة 95 %: 1.32 - 2.15) – فيما يخص الخمول البدني – إلى 48.53 (فاصل الثقة 95 %: 31.98 - 76.65) – فيما يخص محاولة الانتحار. وأشارت النمذجة إلى أن توقي خبرات الطفولة السيئة قد يحد بدرجة كبيرة من وقوع العديد من السلوكيات الضارة بالصحة داخل فئة الدراسة.

**الاستنتاج:**

تشير نتائجنا إلى أنه من الأرجح أن يتمتع الأشخاص الذين لا يكتسبون سلوكيات ضارة بالصحة بطفولة تتميز بالأمان والرعاية. ويتعين نشر البرامج المستندة إلى البيّنات على نطاق واسع في أوروبا الشرقية بغية تحسين إعداد الآباء لرعاية الأبناء ودعم نمو الأطفال.

## 摘要

**目的:**

评价八个东欧国家虐待、忽视、家庭暴力和父母分离、吸毒、精神疾病或监禁等儿童期不良经历与年轻人健康之间的联系。

**方法:**

在2010年至2013年之间，在阿尔巴尼亚、拉脱维亚、立陶宛、黑山共和国、罗马尼亚、俄罗斯联邦、前南斯拉夫马其顿共和国和土耳其展开儿童期不良经历调查。共有10696名18-25岁之间的受访者，其中女性占59.7%。使用多变量模型来研究儿童期不良经历与成年早期危害健康的行为的关系，包括吸毒、缺少运动和自杀企图。

**结果:**

超过半数的受访者表示至少有一次儿童期不良经历。有一次儿童期不良经历增加遭受其他儿童期不良经历的概率。儿童期不良经历数量与危害健康行为的后续报告呈正相关。较之没有报告不良经历的受访者，报告至少有四个儿童期不良经历的受访者其许多危害行为的风险显著增加，优势比从缺乏锻炼的1.68（95%置信区间（CI）：1.32–2.15）到自杀企图的48.53（95% CI：31.98–76.65）。模型表明，预防儿童期不良经历将会大幅度减少研究人群中许多危害健康行为的发生。

**结论:**

我们的结果表明，没有养成危害健康行为的个体更可能是渡过安全、受到关爱的童年。需要在东欧大规模部署以证据为基础的项目，以改善教育和支持孩子发展。

## Резюме

**Цель:**

Оценить связь негативных впечатлений в детстве — например, плохое обращение, пренебрежение, насилие в семье, расставание родителей, употребление психоактивных веществ, психические заболевания или лишение свободы — со здоровьем молодых людей в восьми восточноевропейских странах.

**Методы:**

В 2010-2013 гг. в таких странах, как Албания, Латвия, Литва, Черногория, Румыния, Российская Федерация, бывшая югославская Республика Македония и Турция были проведены опросы среди молодежи, посвященные негативным впечатлениям в детстве. В опросах принимали участие 10 696 респондентов, включая 59,7% девушек в возрасте 18–25 лет. Исследование взаимосвязи между негативными впечатлениями в детстве и вредным для здоровья поведением в старшем подростковом возрасте, в том числе употребление психоактивных веществ, отсутствие физической активности и попытки самоубийства, проводилось методом многомерного моделирования.

**Результаты:**

Более половины респондентов сообщили по меньшей мере об одном негативном впечатлении в детстве. Наличие одного негативного впечатления увеличивало вероятность наличия и последующих негативных впечатлений в детстве. Число негативных впечатлений в детстве положительно коррелирует с последующими сообщениями о вредном для здоровья поведении. По сравнению с теми, кто не переживал негативных впечатлений в детстве, у респондентов, указавших по меньшей мере четыре случая негативных впечатлений, имелся значительно больший риск вредного для здоровья поведения с отношением рисков от 1,68 (95%-й доверительный интервал, ДИ: 1,32–2,15) для физической неактивности и до 48,53 (95% ДИ: 31,98–76,65) для попыток самоубийства. Моделирование показало, что предотвращение негативных впечатлений в детстве существенно уменьшает возникновение многих случаев пагубного поведения внутри исследуемой популяции.

**Вывод:**

Результаты показывают, что лица, у которых не развивается вредное для здоровья поведение, скорее всего имели благополучное детство и родительскую заботу. В восточноевропейских странах требуется масштабное развертывание доказательно обоснованных программ по улучшению семейного воспитания и поддержке развития детей.

## Introduction

The United Nations Convention on the Rights of the Child requires all Member States to offer effective child protection.^1^ Along with a moral imperative for governments to ensure that children are safe and secure, evidence shows the long-term educational, employment and health benefits that can result from protecting children from maltreatment and facilitating supportive parent–child relationships.^2^^–^^4^ Violence has a direct impact on a child’s health through physical and mental injury and, in the most severe cases, death.^5^ However, a child who survives abuse is also at increased risk of developing health-harming and antisocial behaviours during adolescence and noncommunicable conditions, mental illness and disability during adulthood.^6^^–^^8^ The magnitude of these effects is substantive. For example, 30% of the adult mental illnesses identified through World Mental Health Surveys in 21 countries were attributed to physical abuse in childhood or other adverse childhood experiences.^9^

Adverse childhood experiences such as abuse and neglect have an immediate impact upon children and are associated with poorer health and behavioural outcomes. Others such as domestic violence affect the environment around children.^6^^,^^7^ Various tools have been developed to collect data from adults on their childhood experiences and current health status.^10^^–^^12^ Such tools have helped identify relationships between adverse childhood experiences and subsequent sexual risk-taking, substance use and development of health conditions such as obesity, ischaemic heart disease and cancer.^6^^–^^8^^,^^13^^,^^14^

Globally, a higher incidence of child abuse appears to be found in countries with relatively low per-capita incomes.^4^ In many countries there is limited surveillance of childhood abuse and neglect and often an absence of relevant longitudinal studies. Adverse childhood experience surveys can provide rapid empirical data linking childhood experiences with adult health to inform the economic case for – and development of – appropriate interventions early in life.^8^ Consequently, several international health organizations have invested in improving and standardizing tools and methods to investigate adverse childhood experiences.^10^^–^^12^^,^^15^

A life course approach to health has been made a priority for Europe.^16^ A recent meta-analysis of data collected on European children estimated 134 cases of sexual abuse per 1000 girls, 229 cases of physical abuse and 291 cases of emotional abuse per 1000 children.^4^ However, only about one in every three national ministries of health in Europe routinely provides official statistics on child maltreatment.^4^ Within the World Health Organization’s (WHO’s) European Region, levels of child mortality and morbidity appear to be higher in the east than in the west.^4^ Therefore several countries in eastern Europe have undertaken adverse childhood experience surveys, using standardized methods. Here, we use the combined data from these surveys to measure the association between adverse childhood experiences and health-harming behaviours in young adults and to explore how the relationships between such experiences and behaviours vary between the surveyed countries.

## Methods

Health ministries in Albania, Latvia, Lithuania, Montenegro, Romania, the Russian Federation, The former Yugoslav Republic of Macedonia and Turkey (Table 1) undertook adverse childhood experience surveys among young adults (Box 1, available at: http://www.who.int/bulletin/volumes/92/9/13-129247). Each survey was coordinated via the relevant national programme lead for violence prevention. The respondents, who were all in secondary or higher education, were not intended to be representative of the young adults in each country. The adverse childhood experience questionnaire was chosen because it produces data on temporally separated events relatively quickly and is a proven method with validated tools.^10^^,^^12^ It also avoids some problems associated with prospective studies – e.g. the under-identification of maltreatment in child protection data and confounding from the respondents’ maltreatment during the survey process.^17^^,^^18^

**Table 1:** Survey characteristics, outcome variables and missing data, in eight eastern European countries, 2010–2013

Characteristic	Study country								
	All	Albania	Latvia	Lithuania	Montenegro	Romania	Russian Federation	The former Yugoslav Republic of Macedonia	Turkey
Survey dates		February 2012–March 2012	October 2010–March 2011	May 2010–June 2010	October 2012–December 2012	May 2012–June 2012	June 2012–December 2012	March 2010	June 2012–April 2013
Survey setting		Universities	Secondary and vocational schools	Universities and colleges	Universities	Universities	Secondary schools, vocational academies and universities	Secondary schools and universities	Universities
Sampling		Selected universities stratified by academic year. Random class selection	Locations opportunistic, stratification by school type, study language and gender	Random sample of institutes. Opportunistic sample of students	Universities, sample stratified by faculty and then gender	Sample weighted by regional population, random selection of institutions then students in regions	Random sample of schools. Random selection of institutes then students in regions	Random sample of schools then classrooms. Universities random class selection	Selected universities, random sample of faculties and then students
Compliance, %		96	75–100^a^	75–100^a^	98	92	86	90	89
Age, years		18–38	17–25	18–39	18–50	14–66	13–41	17–45	18–41
Cities or areas sampled		Tirana, Vlora, Shkodra and Elbasan	Riga, Liepaja, Jelgava, Cesis, Daugavpils	Vilnius, Kaunas, Panevėžys, Šiauliai, Klaipėda	Podgorica, Bar, Budva, Kotor, Igalo, Nikšić, Bijelo Polje	Bucharest, Central, North-East, North-West, West, South-East, South-West region	Moscow, Volgograd, Republic of Tuva, Republic of Buryatia, Khabarovsk	Skopje, Bitola, Tetovo, Struga, Štip, Gostivar	Ankara, Antalya, Izmir, Trabzon, Van
No. of respondents	13 173	1 437	1 223	1 746	1 565	2 088	1 580	1 277	2 257
No. (%) of respondents aged 18–25 years	12 308	1 395	1 190	1 726	1 501	1 655	1 403	1 227	2 211

**Missing data: events, no. (%)**									
Physical abuse	106 (0.9)	0 (0)	10 (0.8)	30 (1.7)	25 (1.7)	11 (0.7)	0 (0)	4 (0.3)	26 (1.2)
Problematic alcohol use by household member	104 (0.8)	0 (0)	16 (1.3)	15 (0.9)	51 (3.4)	8 (0.5)	0 (0)	4 (0.3)	10 (0.5)
Domestic violence towards mother	133 (1.1)	0 (0)	5 (0.4)	49 (2.8)	33 (2.2)	18 (1.1)	0 (0)	6 (0.5)	22 (1.0)
Parents separated or divorced	89 (0.7)	0 (0)	10 (0.8)	30 (1.7)	24 (1.6)	4 (0.2)	0 (0)	8 (0.7)	13 (0.6)
Emotional neglect	401 (3.3)	0 (0)	23 (1.9)	111 (6.4)	123 (8.2)	30 (1.8)	2 (0.1)	10 (0.8)	104 (4.7)
Depressed or suicidal household member	55 (0.4)	0 (0)	4 (0.3)	19 (1.1)	22 (1.5)	4 (0.2)	0 (0)	2 (0.2)	4 (0.2)
Emotional abuse	262 (2.1)	0 (0)	8 (0.7)	48 (2.8)	162 (10.8)	10 (0.6)	0 (0)	11 (0.9)	23 (1.0)
Sexual abuse	1 072 (8.7)	0 (0)	140 (11.8)	255 (14.8)	248 (16.5)	86 (5.2)	0 (0)	17 (1.4)	326 (14.7)
Household member incarcerated	74 (0.6)	0 (0)	7 (0.6)	23 (1.3)	27 (1.8)	5 (0.3)	0 (0)	4 (0.3)	8 (0.4)
Drug abuse by household member	129 (1.0)	0 (0)	20 (1.7)	25 (1.4)	67 (4.5)	5 (0.3)	0 (0)	5 (0.4)	7 (0.3)
Answering all ACE questions	10 696 (86.9)	1 395 (100.0)	1 003 (84.3)	1 361 (78.9)	1 046 (69.7)	1 527 (92.3)	1 401 (99.9)	1 200 (97.8)	1 763 (79.7)
**Missing data: behaviour, no. (%)**									
Smoker	93 (0.9)	0 (0)	8 (0.8)	36 (2.6)	17 (1.6)	26 (1.7)	0 (0)	1 (0.1)	5 (0.3)
Physical inactivity	57 (0.5)	NA	43 (4.3)	2 (0.1)	4 (0.4)	8 (0.5)	0 (0)	0 (0.0)	NA
Five or more sexual partners	435 (4.1)	0 (0)	132 (13.2)	114 (8.4)	101 (9.7)	81 (5.3)	0 (0)	7 (0.6)	NA
Sexual intercourse at age of less than 16 years	268 (2.5)	3 (0.2)	77 (7.7)	84 (6.2)	58 (5.5)	44 (2.9)	1 (0.1)	1 (0.1)	NA
Drug abuse	92 (0.9)	0 (0)	2 (0.2)	2 (0.1)	9 (0.9)	9 (0.6)	0 (0)	2 (0.2)	68 (3.9)
Problematic use of alcohol	82 (0.8)	0 (0)	NA	0 (0.0)	8 (0.8)	7 (0.5)	0 (0)	0 (0.0)	67 (3.8)
Attempted suicide	44 (0.4)	0 (0)	7 (0.7)	17 (1.2)	5 (0.5)	15 (1.0)	0 (0)	0 (0.0)	NA

ACE: advanced childhood experience; NA: not available.

^a^ Range provided by national coordinators.

Box 1 **Adverse childhood experiences during the first 18 years of life: questions and variation in questions by country**

**Parents separated or divorced**

An affirmative response to the question “were your parents ever separated or divorced?” was considered positive for this adverse childhood experience.

**Domestic violence towards mother**

Based on four questions on domestic violence by father, stepfather or mother’s boyfriend towards mother or stepmother:

- Did he sometimes, often or very often, kick, bite, hit her with a fist or hit her with something hard?
- Did he sometimes, often or very often, push, grab or slap her or throw something at her?
- Did he ever repeatedly hit her for a few minutes or more?
- Did he ever threaten her with a knife or gun?

An affirmative response to any of the questions was considered positive. Respondents in Turkey were asked about domestic violence committed by either parent.

**Emotional neglect**

Based on four statements:

- There was someone in my family who helped me feel important or special.
- I felt loved.
- People in my family looked out for each other.
- You knew there was someone to take care of you and protect you.

Respondents were asked to score each statement from 1 (very often true) to 5 (never true) and those whose total score exceeded 11 were considered positive for emotional neglect.

**Depressed or suicidal household member**

An affirmative response to the question “did you live with a household member who was depressed, mentally ill or suicidal?” was considered positive.

**Physical abuse**

Based on two or four questions:

- Did a household member sometimes, often or very often, push, grab, shove or throw something at you?
- Did a household member ever hit you so hard you had marks or were injured?
- Did a household member ever spank you often or very often, at medium to very hard severity?
- Did a household member spank you sometimes – or a few times a year – quite or very hard?

An affirmative response to any of the questions was considered positive. In Albania, Latvia, Romania and Turkey, only the first two questions were asked.

**Emotional abuse**

Based on one or two questions:

- Did a parent or household member swear at you, insult you, or put you down often or very often?
- Did a parent or household member sometimes, often or very often, act in a way that made you think that you might be physically hurt?

An affirmative response to either question was considered positive. Only the first of the questions was asked in Turkey

**Sexual abuse**

Based on four questions:

- Did an adult or someone at least 5 years older than you touch or fondle your body in a sexual way?
- Did an adult or someone at least 5 years older than you have you touch or fondle their body in a sexual way?
- Did an adult or someone at least 5 years older than you attempt to have any type of sex with you?
- Did an adult or someone at least 5 years older than you have any type of sexual intercourse with you?

A respondent who answered yes to any of the questions was considered positive for this adverse experience if they were reporting any sexual experiences that occurred before they were 16 years of age or non-consensual experiences that occurred before they were 18 years of age.

**Household member incarcerated**

Turkish respondents who answered yes to the question “was anyone in your family imprisoned?” and other respondents who answered yes to the question “did you live with a household member who went to prison?” were considered positive for this adverse childhood experience.

**Problematic use of alcohol by household member**

A respondent who answered yes to the question “did you live with a household member who was a problem drinker or alcoholic?” was considered positive for this adverse childhood experience.

**Drug abuse by household member**

Turkish respondents who answered yes to the question “did you share your house with a drug addict?” and other respondents who answered yes to the question “did you live with a household member who used street drugs?” were considered positive for this adverse childhood experience.

Study coordinators recruited respondents through colleges of further education, universities and schools – including vocational schools (Table 1). The family health history questionnaire developed by the United States Centers for Disease Control and Prevention^10^ was used to measure childhood adversities and health-harming behaviours. However, outcome variables were sometimes changed by study coordinators, to ensure that the measured risks were pertinent to the target populations (Box 2, available at: http://www.who.int/bulletin/volumes/92/9/13-127247). Following translation of the questionnaire and piloting to assess the questionnaire’s validity in each country, the surveys took place in classrooms between May 2010 and April 2013. Sampling used both stratified and random-sample methods, with classes as the sampling unit and each student in attendance asked to complete the questionnaire. Compliance in each country was over 75% (Table 1).

Box 2 **Outcome variables: questions and variation in questions by country**

**Smoker**

A respondent who answered yes to the question “do you smoke currently?” or, in Turkey, “have you ever been a smoker?” was considered to be a smoker.

**Physical inactivity**

A respondent who answered zero to the question “during the past month, about how many days per week did you exercise for recreation or to keep in shape?” or, in Latvia, “over the past 7 days, on how many days were you physically active for a total of at least 60 minutes per day?” was considered to be physically inactive. No data on this topic were collected in Albania and Turkey.

**Five or more sexual partners**

Respondents were asked “with how many different partners have you ever had sexual intercourse?” No data on this topic were collected in Turkey.

**Sexual intercourse at an age of less than 16 years**

Respondents were asked “how old were you the first time you had sexual intercourse?” No data on this topic were collected in Turkey.

**Drug abuse**

A respondent who answered yes to the question “have you ever used street drugs?” or, in Montenegro “have you ever used drugs?” was considered to be a drug abuser.

**Problem drinker or alcoholic**

A respondent who answered yes to the questions: “have you ever had a problem with your use of alcohol?”, “have you ever considered yourself to be an alcoholic?” or, in Turkey, “do you sometimes drink more than is good for you?” was considered to be a problematic user of alcohol. No data on this topic were collected in Latvia.

**Attempted suicide**

Respondents in all the study countries except Turkey were asked “have you ever attempted to commit suicide?”

For our multi-study analyses, data were limited to those from the 94.3% (12 308/13 173) of respondents who were aged 18–25 years (Table 1). Most of the questions on adverse childhood experiences and health-harming behaviours were completed by over 95% of these respondents. However, the questions on sexual abuse were only completed by 91.3% (11 236/12 308) of respondents. There was between-country variation in the percentages of respondents who answered individual questions. For example; questions about emotional abuse were completed by all of the respondents in Albania and the Russian Federation but only by 89.2% (1339/1501) of the respondents in Montenegro (Table 1). Analysis was limited to respondents that provided information on all 10 adverse childhood experience categories (86.9%, 10 696/12 308; Table 1). Such respondents had a mean age of 20.1 years and were more likely to be female (59.7%, 6389/10 696) than male (Table 2). Parental educational attainment was used as a proxy for the respondents’ socioeconomic status when they were children.^19^ This attainment was categorized as high if the parent had graduated from a college of further education or university, middle if the parent had completed secondary or technical school education or had enrolled in – but not completed – a college course and low if the parent had no education, was illiterate, had attended mandatory studies only, had only received an elementary or primary education or had attended a secondary school but had not completed the course. Father’s educational attainment was used for the analyses wherever available (*n* = 10 392). Otherwise, the mother’s attainment was used (*n* = 224) or, if neither parent’s attainment was known (*n* = 80), the parental attainment was assumed to be middle. The outcomes examined were those that had been measured in at least five of the study countries (Box 2).

**Table 2:** Sample demographics and proportions with adverse childhood experiences by country, in eight eastern European countries, 2010–2013

Characteristic	Study country								
	All (*n* = 10 696)	Albania (*n* = 1 395)	Latvia (*n* = 1 003)	Lithuania (*n* = 1 361)	Montenegro (*n* = 1 046)	Romania (*n* = 1 527)	Russian Federation (*n* = 1 401)	The former Yugoslav Republic of Macedonia (*n* = 1 200)	Turkey (*n* = 1 763)
**Sex, no. (%)**									
Female	6 389 (59.7)	943 (67.6)	511 (50.9)	922 (67.7)	545 (52.1)	957 (62.7)	840 (60.0)	718 (59.8)	953 (54.1)
Male	4 307 (40.3)	452 (32.4)	492 (49.1)	439 (32.3)	501 (47.9)	570 (37.3)	561 (40.0)	482 (40.2)	810 (45.9)
**Age group, no. (%)**									
18–19 years	4 480 (41.9)	304 (21.8)	906 (90.3)	477 (35.0)	593 (56.7)	228 (14.9)	459 (32.8)	720 (60.0)	793 (45.0)
20–21 years	4 237 (39.6)	608 (43.6)	77 (7.7)	820 (60.2)	379 (36.2)	734 (48.1)	554 (39.5)	344 (28.7)	721 (40.9)
22–25 years	1 979 (18.5)	483 (34.6)	20 (2.0)	64 (4.7)	74 (7.1)	565 (37.0)	388 (27.7)	136 (11.3)	249 (14.1)
**Level of parental education, no. (%)**									
Low	1 295 (12.1)	187 (13.4)	56 (5.6)	30 (2.2)	46 (4.4)	139 (9.1)	181 (12.9)	115 (9.6)	541 (30.7)
Medium	5 658 (52.9)	714 (51.2)	601 (59.9)	689 (50.6)	672 (64.2)	929 (60.8)	711 (50.7)	747 (62.2)	595 (33.7)
High	3 743 (35.0)	494 (35.4)	346 (34.5)	642 (47.2)	328 (31.4)	459 (30.1)	509 (36.3)	338 (28.2)	627 (35.6)
**Events reported, no. (%)**^a^									
Physical abuse	1 993 (18.6)	572 (41.0)	162 (16.2)	176 (12.9)	205 (19.6)	357 (23.4)	180 (12.8)	83 (6.9)	258 (14.6)
Problematic use of alcohol by household member	1 753 (16.4)	289 (20.7)	304 (30.3)	357 (26.2)	114 (10.9)	341 (22.3)	128 (9.1)	108 (9.0)	112 (6.4)
Domestic violence towards mother	1 563 (14.6)	419 (30.0)	204 (20.3)	225 (16.5)	96 (9.2)	103 (6.7)	183 (13.1)	17 (1.4)	316 (17.9)
Parents separated or divorced	1 508 (14.1)	92 (6.6)	424 (42.3)	270 (19.8)	99 (9.5)	250 (16.4)	235 (16.8)	45 (3.8)	93 (5.3)
Emotional neglect	1 257 (11.8)	227 (16.3)	89 (8.9)	137 (10.1)	77 (7.4)	116 (7.6)	258 (18.4)	200 (16.7)	153 (8.7)
Depressed or suicidal household member	1 069 (10.0)	106 (7.6)	189 (18.8)	140 (10.3)	59 (5.6)	211 (13.8)	115 (8.2)	82 (6.8)	167 (9.5)
Emotional abuse	858 (8.0)	370 (26.5)	79 (7.9)	59 (4.3)	49 (4.7)	136 (8.9)	40 (2.9)	59 (4.9)	66 (3.7)
Sexual abuse	798 (7.5)	266 (19.1)	70 (7.0)	47 (3.5)	38 (3.6)	89 (5.8)	78 (5.6)	89 (7.4)	121 (6.9)
Household member incarcerated	567 (5.3)	52 (3.7)	83 (8.3)	48 (3.5)	75 (7.2)	35 (2.3)	77 (5.5)	50 (4.2)	147 (8.3)
Drug abuse by household member	276 (2.6)	21 (1.5)	48 (4.8)	20 (1.5)	32 (3.1)	36 (2.4)	20 (1.4)	43 (3.6)	56 (3.2)
**Event count per respondent, no. (%)**									
0	5 068 (47.4)	422 (30.3)	281 (28.0)	644 (47.3)	593 (56.7)	709 (46.4)	699 (49.9)	721 (60.1)	999 (56.7)
1	2 669 (25.0)	317 (22.7)	288 (28.7)	335 (24.6)	251 (24.0)	400 (26.2)	389 (27.8)	299 (24.9)	390 (22.1)
2	1 398 (13.1)	236 (16.9)	187 (18.6)	195 (14.3)	114 (10.9)	202 (13.2)	162 (11.6)	109 (9.1)	193 (10.9)
3	771 (7.2)	227 (16.3)	106 (10.6)	87 (6.4)	41 (3.9)	99 (6.5)	75 (5.4)	43 (3.6)	93 (5.3)
> 3	790 (7.4)	193 (13.8)	141 (14.1)	100 (7.3)	47 (4.5)	117 (7.7)	76 (5.4)	28 (2.3)	88 (5.0)

^a^ The reported proportions of adverse childhood experiences should not be compared directly because the sampling process differed between countries.

Version 18 of PASW Statistics (SPSS Inc., Chicago, United States of America) and version 2.7.9 of StatsDirect (StatsDirect, Altrincham, England) were used for the basic analyses and multinational meta-analysis, respectively. Dependent variables were dichotomized where necessary and relationships between individual adverse childhood experiences were examined using *χ^2^* analyses. Having any adverse childhood experience was highly positively associated with having any other adverse childhood experience (*P* < 0.001). Thus, as in previous adverse childhood experience studies,^6^^–^^8^ the number of adverse childhood experience types reported by a respondent – the adverse childhood experience count – was categorized as 0, 1, 2, 3 or greater than 3 (Table 2). To control for independent associations between demographics and differences in outcome results between countries, hierarchical binomial logistic regression was used, with country in the first stratum. A hierarchical model was chosen to account for potential dependencies between individual observations introduced through sampling in different countries.^20^ The model-derived probabilities of health-harming behaviours associated with demographics and adverse childhood experience counts were used to generate an expected sample prevalence for each health-harming behaviour if all of the respondents’ adverse childhood experience counts had been zero. As well as the multinational logistic regression models, models were generated for each outcome in each study country. Using the adjusted odds ratios (aORs) calculated from the country-level models, data were pooled across studies using the summary meta-analysis function in StatsDirect. For Albania and Montenegro there were insufficient individuals with more than three adverse childhood experiences and attempted suicide to generate country level odds ratios. The same was true for drug abuse in the Albania sample. With zero adverse childhood experiences used as the reference category, two statistics – *I^2^* and Cochran’s *Q* – were used to estimate the between-country heterogeneity in the association between having an adverse childhood experience count above 3 and each of the health-harming behaviours. Forest plots were generated to show the aOR and corresponding 95% confidence interval (CI) for each behaviour – for each study country and for the pooled data. Ethical approval was obtained separately within each study country, using the appropriate processes. Ethical approval for the analysis of combined data from different countries was provided by the Research Ethics Committee of Liverpool John Moores University.

## Results

Over half of the respondents reported at least one adverse childhood experience each. The most commonly reported adverse childhood experience was physical abuse (Table 2). In most countries, the reported prevalence of sexual abuse, emotional abuse and having a household member who was incarcerated or used drugs were relatively low. However, sexual abuse was reported by 7.5% (798/10 696) of the respondents. The between-country variation in levels of each type of adverse childhood experience was statistically significant (*P* < 0.05). The study countries also differed in terms of the respondents’ demographics (Table 2) and their reported health-harming behaviours (Table 3).

**Table 3:** Percentage of respondents^a^ reporting health-harming behaviours by country, in eight eastern European countries, 2010–2013

Behaviour	Percentage of respondents (no. HHB/*n)*								
	Overall	Albania	Latvia	Lithuania	Montenegro	Romania	Russian Federation	The former Yugoslav Republic of Macedonia	Turkey
Smoker	27.2 (2 888/10 603)	22.0 (307/1 395)	40.5 (403/995)	32.5 (431/1 325)	16.3 (168/1 029)	32.9 (494/1 501)	21.2 (297/1 401)	26.9 (323/1 199)	26.5 (465/1 758)
Physical inactivity	17.0 (1 274/7 481)	NA	5.5 (53/960)	17.0 (231/1 359)	26.7 (278/1 042)	20.6 (313/1 519)	16.1 (225/1 401)	14.5 (174/1 200)	NA
Five or more sexual partners	14.0 (1 186/8 498)	9.2 (129/1 395)	16.2 (141/871)	11.1 (138/1 247)	16.7 (158/945)	20.9 (302/1 446)	15.5 (217/1 401)	8.5 (101/1 193)	NA
Sexual intercourse when aged < 16 years	12.8 (1 107/8 665)	13.3 (185/1 392)	21.9 (203/926)	5.5 (70/1 277)	9.8 (97/988)	12.1 (180/1 483)	19.5 (273/1 400)	8.3 (99/1 199)	NA
Drug abuse	12.0 (1 271/10 604)	4.1 (57/1 395)	27.7 (277/1 001)	29.2 (397/1 359)	10.5 (109/1 037)	15.2 (230/1 518)	4.8 (67/1 401)	5.4 (65/1 198)	4.1 (69/1 695)
Problematic use of alcohol	9.2 (885/9 611)	12.0 (168/1 395)	NA	9.0 (123/1 361)	8.5 (88/1 038)	7.2 (109/1 520)	10.5 (147/1 401)	6.5 (78/1 200)	10.1 (172/1 696)
Attempted suicide	4.1 (364/8 889)	3.6 (50/1 395)	6.3 (63/996)	4.6 (62/1 344)	2.7 (28/1 041)	4.4 (66/1 512)	4.2 (59/1 401)	3.0 (36/1 200)	NA

HHB: health-harming behaviour; NA: not available.

^a^ The percentage of respondents reporting health-harming behaviours should not be compared directly because the sampling process differed between countries.

All types of adverse childhood experience were significantly associated with smoking, the problematic use of alcohol and drug abuse (Table 4). Compared with the other respondents, those who had lived with a drug abuser in childhood were much more likely to have used drugs themselves (44.9% [122/272] versus 11.1% [1149/10 332]) and those who had lived with a problematic user of alcohol were more likely to report problematic use of alcohol themselves (24.0% [347/1444] versus 6.6% [538/8167]). The proportion of respondents who reported that they had attempted suicide reached 18.5% (166/896) among those who had lived with someone who was depressed or suicidal – compared with just 2.5% (198/7993) among the other respondents. Further, respondents who reported that they were sexually abused as children were much more likely to report that they had had sexual intercourse when aged less than 16 years (31.2% [208/666] versus 11.2% [899/7999]; Table 4).

**Table 4:** Proportions reporting each health-harming behaviour by exposure to individual adverse childhood experiences, in eight eastern European countries, 2010–2013

Adverse childhood experience	Health-harming behaviour																			
	Smoker (*n* = 10 603)			Physical inactivity (*n* = 7 481)^a^			At least five sexual partners (*n* = 8 498)^a^			Sexual intercourse when aged < 16 years (*n* = 8 665)^b^			Drug abuse (*n* = 10 604)			Problematic use of alcohol (*n* = 9 611)^c^			Attempted suicide (*n* = 8 889)^b^	
	% (no. HHB/no. ACE)	*P*		% (no. HHB/no. ACE)	*P*		% (no. HHB/no. ACE)	*P*		% (no. HHB/no. ACE)	*P*		% (no. HHB/no. ACE)	*P*		% (no. HHB/no. ACE)	*P*		% (no. HHB/no. ACE)	*P*
**Physical abuse**																				
No	25.5 (2 201/8 619)			16.4 (1 035/6 328)			12.5 (850/6 825)			11.6 (810/6 968)			10.7 (926/8 626)			7.5 (584/7 794)			2.6 (183/7 163)	
Yes	34.6 (687/1 984)	< 0.001		20.7 (239/1 153)	< 0.001		20.1 (336/1 673)	< 0.001		17.5 (297/1 697)	< 0.001		17.4 (345/1 978)	< 0.001		16.6 (301/1 817)	< 0.001		10.5 (181/1 726)	< 0.001
**Problematic use of alcohol by household member**																				
No	25.1 (2 222/8 867)			16.4 (1 010/6 150)			13.6 (940/6 934)			12.5 (887/7 072)			9.9 (876/8 860)			6.6 (538/8 167)			2.6 (192/7 262)	
Yes	38.4 (666/1 736)	< 0.001		19.8 (264/1 331)	< 0.01		15.7 (246/1 564)	< 0.05		13.8 (220/1 593)	NS		22.6 (395/1 744)	< 0.001		24.0 (347/1 444)	< 0.001		10.6 (172/1 627)	< 0.001
**Domestic violence towards mother**																				
No	26.4 (2 390/9 046)			16.6 (1 103/6 661)			13.7 (1 004/7 306)			12.2 (911/7 448)			11.4 (1 032/9 061)			8.1 (671/8 267)			3.0 (227/7 648)	
Yes	32.0 (498/1 557)	< 0.001		20.9 (171/820)	< 0.01		15.3 (182/1 192)	NS		16.1 (196/1 217)	< 0.001		15.5 (239/1 543)	< 0.001		15.9 (214/1 344)	< 0.001		11.0 (137/1 241)	< 0.001
**Parents separated or divorced**																				
No	25.1 (2 290/9 110)			16.8 (1 036/6 176)			12.9 (920/7 159)			11.7 (856/7 295)			9.8 (895/9 103)			8.3 (712/8 533)			2.8 (211/7 485)	
Yes	40.1 (598/1 493)	< 0.001		18.2 (238/1 305)	NS		19.9 (266/1 339)	< 0.001		18.3 (251/1 370)	< 0.001		25.0 (376/1 501)	< 0.001		16.0 (173/1 078)	< 0.001		10.9 (153/1 404)	< 0.001
**Emotional neglect**																				
No	26.5 (2 478/9 358)			16.7 (1 105/6 611)			13.2 (983/7 447)			12.2 (923/7 592)			11.6 (1 088/9 359)			8.3 (703/8 454)			3.2 (246/7 792)	
Yes	32.9 (410/1245)	< 0.001		19.4 (169/870)	< 0.05		19.3 (203/1 051)	< 0.001		17.1 (184/1 073)	< 0.001		14.7 (183/1 245)	< 0.01		15.7 (182/1 157)	< 0.001		10.8 (118/1 097)	< 0.001
**Depressed or suicidal household member**																				
No	26.0 (2 485/9 546)			16.5 (1 102/6 693)			13.8 (1 053/7 633)			12.5 (975/7 787)			10.7 (1 020/9 548)			8.0 (699/8 738)			2.5 (198/7 993)	
Yes	38.1 (403/1 057)	< 0.001		21.8 (172/788)	< 0.001		15.4 (133/865)	NS		15.0 (132/878)	< 0.05		23.8 (251/1 056)	< 0.001		21.3 (186/873)	< 0.001		18.5 (166/896)	< 0.001
**Emotional abuse**																				
No	26.3 (2 561/9 747)			16.7 (1 181/7 063)			13.3 (1 025/7 728)			12.0 (944/7 885)			11.6 (1 127/9 751)			8.2 (726/8 836)			2.9 (238/8 102)	
Yes	38.2 (327/856)	< 0.001		22.2 (93/418)	< 0.01		20.9 (161/770)	< 0.001		20.8 (162/780)	< 0.001		16.9 (144/853)	< 0.001		20.5 (159/775)	< 0.001		16.0 (126/787)	< 0.001
**Sexual abuse**																				
No	26.4 (2 593/9 808)			17.1 (1 209/7 071)			13.2 (1 039/7 842)			11.2 (899/7 999)			11.6 (1 143/9 818)			8.3 (742/8 895)			3.4 (277/8 214)	
Yes	37.1 (295/795)	< 0.001		15.9 (65/410)	NS		22.4 (147/656)	< 0.001		31.2 (208/666)	< 0.001		16.3 (128/786)	< 0.001		20.0 (143/716)	< 0.001		12.9 (87/675)	< 0.001
**Household member incarcerated**																				
No	26.7 (2 685/10 039)			17.0 (1 211/7 115)			13.8 (1 115/8 107)			12.5 (1 032/8 258)			11.5 (1 156/10 045)			8.7 (792/9 133)			3.2 (268/8 473)	
Yes	36.0 (203/564)	< 0.001		17.2 (63/366)	NS		18.2 (71/391)	< 0.05		18.4 (75/407)	< 0.001		20.6 (115/559)	< 0.001		19.5 (93/478)	< 0.001		23.1 (96/416)	< 0.001
**Drug abuse by household member**																				
No	26.6 (2 745/10 327)			17.0 (1 240/7 283)			13.6 (1 129/8 291)			12.4 (1 052/8 451)			11.1 (1 149/10 332)			8.7 (816/9 388)			3.6 (314/8 670)	
Yes	51.8 (143/276)	< 0.001		17.2 (34/198)	NS		27.5 (57/207)	< 0.001		25.7 (55/214)	< 0.001		44.9 (122/272)	< 0.001		30.9 (69/223)	< 0.001		22.8 (50/219)	< 0.001
**No. of ACE reported by respondent**																				
0	21.1 (1 059/5 017)			16.0 (579/3 625)			10.5 (406/3 865)			9.6 (377/3 934)			7.4 (373/5 027)			4.6 (216/4 747)			0.7 (30/4 051)	
1	29.1 (770/2 647)			16.5 (322/1 949)			15.8 (341/2 159)			12.9 (284/2 210)			12.6 (332/2 645)			9.2 (217/2 360)			2.2 (49/2 268)	
2	30.4 (422/1 386)			17.9 (172/959)			15.2 (174/1 147)			14.8 (173/1 167)			16.5 (228/1 384)			13.8 (165/1 200)			5.2 (62/1 199)	
3	38.4 (295/768)			20.7 (92/445)			16.8 (110/656)			18.0 (121/671)			16.2 (124/765)			15.6 (103/661)			8.7 (59/675)	
> 3	43.6 (342/785)	< 0.001		21.7 (109/503)	< 0.01		23.1 (155/671)	< 0.001		22.3 (152/683)	< 0.001		27.3 (214/783)	< 0.001		28.6 (184/643)	< 0.001		23.6 (164/696)	< 0.001

ACE: advanced childhood experience; HHB: health-harming behaviour; NS: not significant.

^a^ Excludes Turkey and Albania because the relevant data were not collected for these countries.

^b^ Excludes Turkey because the relevant data were not collected for this country.

^c^ Excludes Latvia because the relevant data were not collected for this country.

More than half (53.1%; 6177/11 642) of all adverse childhood experiences reported were experienced by the 14.6% (1561/10 696) of respondents who each reported at least three adverse childhood experiences. Adverse childhood experience counts were positively correlated with each of the health-harming behaviours (Table 4), even after controlling for country and demographic effects. Compared with the respondents who reported no adverse childhood experiences, those who reported at least four adverse childhood experiences had higher odds for health-harming behaviours; from aOR: 1.68 for physical inactivity to aOR: 48.53 for attempted suicide (Table 5).

**Table 5:** Demographics and number of adverse childhood experiences as predictors of health-harming behaviours, in eight eastern European countries,^a^ 2010–2013

Variable	Adjusted odds ratio (95% CI)^b^						
	Smoker	Physical inactivity	At least five sexual partners	Sexual intercourse when aged < 16 years	Drug abuse	Problematic use of alcohol	Attempted suicide
**Sex**							
Female	1.0	1.0	1.0	1.0	1.0	1.0	1.0
Male	1.71 (1.56–1.87)	0.42 (0.36–0.48)	5.99 (5.19–6.92)	4.02 (3.50–4.62)	2.54 (2.23–2.90)	1.93 (1.67–2.23)	0.30 (0.22–0.40)
**Age, years**							
18–19	1.0	1.0	1.0	1.0	1.0	1.0	1.0
20–21	1.07 (0.97–1.19)	1.12 (0.96–1.30)	1.71 (1.44–2.03)	1.04 (0.88–1.23)	1.44 (1.23–1.70)	1.16 (0.98–1.37)	1.01 (0.76–1.34)
22–25	0.99 (0.86–1.13)	1.46 (1.21–1.77)	2.16 (1.77–2.64)	0.87 (0.71–1.06)	1.55 (1.25–1.92)	1.16 (0.94–1.42)	1.11 (0.79–1.58)
**Level of parental education**							
Low	1.0	1.0	1.0	1.0	1.0	1.0	1.0
Medium	1.01 (0.87–1.17)	0.92 (0.73–1.16)	0.91 (0.72–1.16)	1.01 (0.80–1.28)	1.26 (0.97–1.64)	1.11 (0.88–1.41)	0.87 (0.60–1.24)
High	1.09 (0.94–1.28)	0.86 (0.67–1.11)	1.24 (0.97–1.60)	1.25 (0.98–1.60)	1.97 (1.51–2.57)	1.68 (1.32–2.13)	0.91 (0.61–1.34)
**ACE count**							
0	1.0	1.0	1.0	1.0	1.0	1.0	1.0
1	1.48 (1.33–1.65)	1.19 (1.02–1.39)	1.53 (1.30–1.80)	1.25 (1.05–1.48)	1.70 (1.44–2.00)	2.11 (1.73–2.57)	3.30 (2.08–5.23)
2	1.63 (1.42–1.87)	1.28 (1.05–1.55)	1.74 (1.42–2.14)	1.72 (1.40–2.10)	2.54 (2.09–3.07)	3.55 (2.86–4.42)	7.96 (5.09–12.45)
3	2.48 (2.09–2.93)	1.60 (1.24–2.07)	2.26 (1.75–2.91)	2.19 (1.72–2.79)	2.98 (2.34–3.79)	4.21 (3.25–5.46)	15.50 (9.79–24.55)
> 3	3.03 (2.57–3.57)	1.68 (1.32–2.15)	3.67 (2.90–4.64)	3.14 (2.50–3.95)	5.71 (4.61–7.08)	9.74(7.74–12.26)	48.53 (31.98–76.65)

ACE: adverse childhood experience; CI: confidence interval.

^a^ Albania, Latvia, Lithuania, Montenegro, Romania, Russian Federation, The former Yugoslav Republic of Macedonia and Turkey.

^b^ Country of survey was included in the first stratum of the hierarchical binomial logistic regression model. Country effects were significant for all of the health-harming behaviours investigated.

**Fig. 1:**
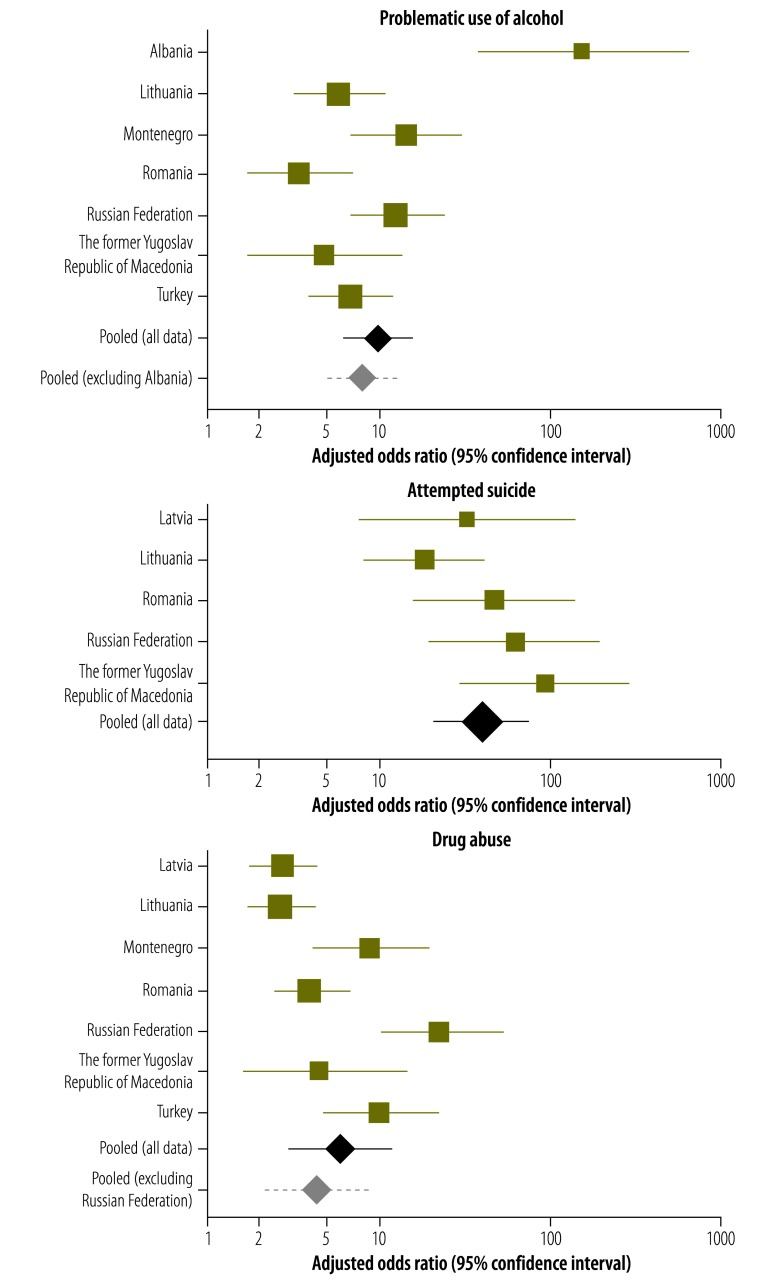
Country-specific likelihood of health-harming behaviours in young adults with at least four adverse childhood experiences, in eight eastern European countries, 2010–2013

Notes: The size of the data points is proportional to the study's weight in the meta-analysis. Adjusted odds ratios were calculated for respondents who reported at least four adverse childhood experiences each. Respondents who reported no such experiences were used as the reference category. We used a binomial logistic regression model with each health-harming behaviour as the dependent and categorized counts of adverse childhood experiences and demographics (age, gender, and parental education level) as independent variables. High odds ratios were generated when particular health-harming behaviours were rare in those with no adverse childhood experiences but relatively frequent in those with four or more adverse childhood experiences.

**Fig. 2:**
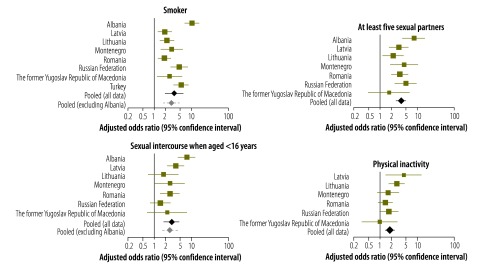
Country-specific likelihood of health-harming behaviours in young adults with at least four adverse childhood experiences, in eight eastern European countries, 2010–2013

Note: The size of the data points is proportional to the study's weight in the meta-analysis. Adjusted odds ratios were calculated for respondents who reported at least four adverse childhood experiences each. Respondents who reported no such experiences were used as the reference category.

We ran separate logistic regression models for each country before using a meta-analysis – and a reference category of a zero adverse childhood experience count – to examine the between-country heterogeneity in the association between an adverse childhood experience count above 3 and health-harming behaviours. Pooled aORs (Fig. 1 and Fig. 2) indicated low between-country heterogeneity (*P* > 0.100) for the relationships between an adverse childhood experience count above 3 and attempted suicide (*I^2^* = 34.7%); having had five or more sexual partners (*I^2^ =* 37.2%); and physical inactivity (*I^2^* = 37.6%). Although there was evidence of substantial between-country heterogeneity in the relationship between an adverse childhood experience count above 3 and having had sexual intercourse when aged less than 16 years (*I^2^* = 66.8%), this was markedly reduced when the outlying data from Albania were excluded (*I^2^* = 15.3%). Albania was also an outlier for the association between such an adverse childhood experience count and the problematic use of alcohol and smoking, although substantial between-country heterogeneity in these relationships remained after the Albanian data were excluded – with *I^2^*  values of  57.0% (*P* = 0.040) and 68.1% (*P* = 0.005), respectively. There was evidence of substantial between-country heterogeneity in the association between an adverse childhood experience count above 3 and drug abuse. The level of heterogeneity in this relationship remained substantial even after the outlying data for the Russian Federation were excluded (*I^2^* = 67.6%; *P* = 0.009). For all of the health-harming behaviours, the random pooled adjusted odds ratios generated in the meta-analysis were consistent with those developed though combined logistic regression (Table 5).

Finally, using the probabilities derived from the logistic regression models (Table 5), we estimated the reduction in levels of each health-harming behaviour if all adverse childhood experiences were eliminated. The percentage of respondents reporting smoking, drug abuse, having at least five sexual partners and having sexual intercourse at an age of less than 16 years would all have been lower – falling from 27.2% (2888/10 603) to 21.3% (2258/10 603), 12.0% (1271/10 604) to 7.7% (817/10 604), 14.0% (1186/8498) to 11.1% (943/8498) and 12.8% (1107/8665) to 10.1% (875/8665), respectively. The greatest reductions would be seen in problematic alcohol use and attempted suicide, which would fall from 9.2% (885/9611) to 4.5% (432/9611) and from 4.1% (364/8889) to 0.7% (62/8889) of respondents respectively.

## Discussion

Our findings from analyses of adverse childhood experience surveys in eight countries show significant association between adverse childhood experiences and health-harming behaviours of young adults who live in the east of WHO’s European Region. The relationship that we observed between the number of adverse childhood experiences and such behaviours are consistent with the results of previous studies.^6^^,^^7^ However, such associations have not been previously examined using a multinational sample from eastern Europe.

While additional studies and meta-analyses are required to confirm and extend our results, it appears that – even in populations with different levels of health-harming behaviours – there are some consistent relationships between such behaviours in young adulthood and adverse childhood experiences. We estimated that if all adverse childhood experience were eliminated, major reductions in health-harming behaviours would follow. Although such reductions provide an indication of the extent to which adverse childhood experiences affect health-harming behaviours, a reduction in the incidence of adverse childhood experiences remains a more realistic objective than their elimination.

In the sample that we investigated, adverse childhood experiences were associated with subsequent health-harming behaviours that were largely independent of the respondent’s age, sex and level of parental education. Three out of the seven behaviours that we investigated – smoking, physical inactivity and attempted suicide – had no independent relationship with the level of parental education. However, many of the respondents were recruited while they were in higher education and this may have introduced bias against more disadvantaged groups. More than half of the respondents had at least one parent who had completed secondary or technical school education. Sampling frameworks also varied between countries. For example, the Latvian respondents came from secondary and vocational schools whereas the Turkish respondents came from colleges of further education and universities. Any bias within and between samples may have affected the reported levels of adverse childhood experiences and health-harming behaviours. Although age, sex, parental education and country were included in our multivariate models to account for such variations, further research is required to evaluate levels of each adverse childhood experience and health-harming behaviour – specifically in disadvantaged groups.

One or two countries appeared to differ from the general trend for certain relationships between adverse childhood experiences and health-harming behaviours. For example, Albania appeared to be an outlier for the relationships between adverse childhood experiences and smoking, sexual intercourse when aged less than 16 years and problematic alcohol use. It is unclear if a high incidence of specific adverse childhood experiences – such as domestic violence – in the Albanian sample affected such relationships and this requires further study. Moreover, we are unable to identify if cultural differences between countries (e.g. tolerance of alcohol) may have impacted on reporting (e.g. problematic alcohol use by respondents or by a member of their childhood household). Although the surveys were based on one standardized questionnaire, minor adaptations to that questionnaire in each country and the questionnaire’s translation from English into each native language may also have introduced bias. Like other investigations on adverse childhood experiences, this study relied on retrospective recall and consequently may have been affected by recall error. While levels of participation in the surveys and completion of most questions were generally high, relatively large numbers of the respondents failed to answer the questions on sexual abuse and sexual partners. It is not possible to identify if differences in the target groups or surveyors affected the completion of individual questions and the study’s main findings. The percentage of respondents who reported that they had been physically abused in childhood (18.6%; 1993/10 696) was similar to an earlier estimate of the prevalence of the physical abuse of European children (22.9%),^4^ which was based on global meta-analyses.^21^^–^^24^ Similarly, the percentage of respondents who reported that they had been sexually abused in childhood (7.5%) was similar to the corresponding values – 13.4% for females and 5.7% for males – reported previously.^4^ In contrast, the prevalence of emotional abuse previously recorded (29.1%)^4^ was markedly higher than the corresponding value that we recorded (8.0%; 858/10 696), perhaps because of between-study variation in the definition of such abuse.

Worldwide, an estimated 28 000 deaths in children aged 0–14 years were due to homicide in 2010.^5^^,^^25^ The actual figure is likely to be substantially higher because of under-reporting and the poor investigation of child deaths in many countries.^26^^,^^27^ For every fatal case of child maltreatment, thousands of children suffer non-fatal maltreatment, much of which will go unreported. For example, in England between 2009 and 2010, there were 62 child maltreatment fatalities and 43 700 child maltreatment cases substantiated through child protection data.^28^ In a different study in the United Kingdom of Great Britain and Northern Ireland, 2.5% of children aged less than 11 years and 6.0% of those aged 11–17 years were found to have experienced physical, sexual or emotional abuse or neglect in the previous year.^29^ These percentages indicated that child maltreatment was 7- to 17-fold more common than recorded by official reports.^29^ In the USA, the lifetime economic burden of the new cases of child maltreatment that were identified in the year 2008 – including the costs of childhood health care, child welfare, special education, criminal justice, adult medical costs and productivity losses – was estimated to be approximately 124 billion United States dollars (US$).^30^ The corresponding estimated costs per victim were US$ 210 012 and US$ 1 272 900 for non-fatal and fatal maltreatment, respectively.^30^ Although the corresponding costs for our eight study countries are not available, they are likely to be substantive given that abuse, neglect and other stressors in early life so frequently lead to health-harming behaviours and, ultimately, to noncommunicable diseases.^9^^,^^31^^–^^33^

A WHO report on preventing child maltreatment in Europe^4^ argues that child maltreatment and other adverse childhood experiences – and their consequences – could be prevented by interdisciplinary approaches. The high burden of adverse childhood experiences and the potential cost–effectiveness of their prevention make a compelling argument for increased investment in adverse childhood experience prevention and for mainstreaming such prevention into many areas of health and social policy.^4^ Unfortunately, the routine collection of data on childhood abuse is rare in Europe. Cross-sector work between health, social, education and criminal justice agencies is also rare, typically small-scale and seldom focuses on the primary prevention of adverse childhood experiences. Cost–effective health and social interventions – e.g. home visiting and parent training – can improve childhood environments and reduce antisocial behaviour in later adolescence.^2^^–^^4^^,^^34^^–^^36^ Adverse childhood experience surveys offer a rapid mechanism to encourage investment in such interventions, by identifying the scale of family problems and the potential health and social benefits of addressing such problems. WHO has called on health ministries to take a leadership role and ensure the development of national policies for the prevention of child maltreatment.^37^
